# Promises and Challenges of populational Proteomics in Health and Disease

**DOI:** 10.1016/j.mcpro.2024.100786

**Published:** 2024-05-17

**Authors:** Benjamin B. Sun, Karsten Suhre, Bradford W. Gibson

**Affiliations:** 1 Human Genetics, Informatics and Predictive Sciences, Bristol-Myers Squibb, Cambridge, Massachusetts, USA; 2 Bioinformatics Core, Weill Cornell Medicine-Qatar, Education City, Doha, Qatar; 3 Englander Institute for Precision Medicine, Weill Cornell Medicine, New York, New York, USA; 4 Pharmaceutical Chemistry, University of California, San Francisco, California, USA

**Keywords:** proteomics, genomics, biobank, genetic association studies, pQTL, mass spectrometry, affinity-based proteomics, high-throughput proteomics, population cohorts, risk prediction, multiplex proteomics

## Abstract

Advances in proteomic assay technologies have significantly increased coverage and throughput, enabling recent increases in the number of large-scale population-based proteomic studies of human plasma and serum. Improvements in multiplexed protein assays have facilitated the quantification of thousands of proteins over a large dynamic range, a key requirement for detecting the lowest-ranging, and potentially the most disease-relevant, blood-circulating proteins. In this perspective, we examine how populational proteomic datasets in conjunction with other concurrent omic measures can be leveraged to better understand the genomic and non-genomic correlates of the soluble proteome, constructing biomarker panels for disease prediction, among others. Mass spectrometry workflows are discussed as they are becoming increasingly competitive with affinity-based array platforms in terms of speed, cost, and proteome coverage due to advances in both instrumentation and workflows. Despite much success, there remain considerable challenges such as orthogonal validation and absolute quantification. We also highlight emergent challenges associated with study design, analytical considerations, and data integration as population-scale studies are run in batches and may involve longitudinal samples collated over many years. Lastly, we take a look at the future of what the nascent next-generation proteomic technologies might provide to the analysis of large sets of blood samples, as well as the difficulties in designing large-scale studies that will likely require participation from multiple and complex funding sources and where data sharing, study designs, and financing must be solved.

## Highlights

• Common uses of large-scale population proteomics in health and disease.
• Broad synopsis of main types of scalable proteomic methods.
• Overview of large proteomic and proteogenomic studies in human blood.
• Summary of key challenges and future outlook of populational proteomics.

## Graphical Abstract

**Figure:**
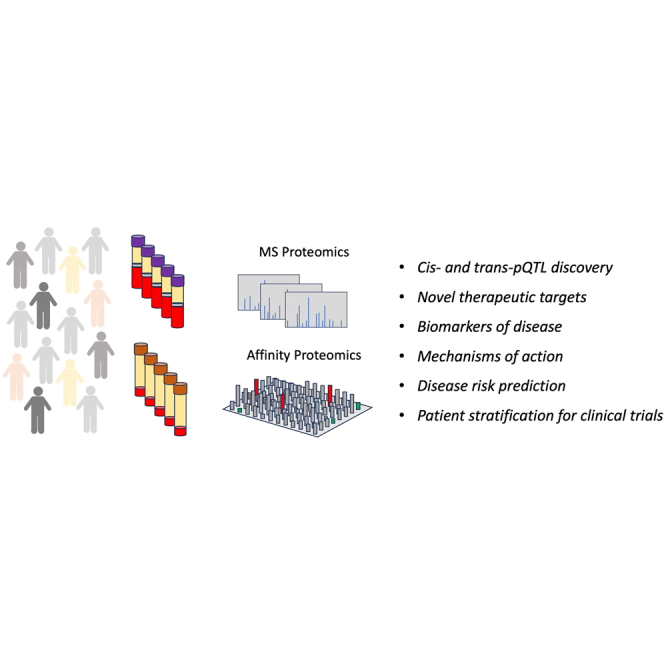

## In Brief

Populational cohort-based proteomics assays across thousands of proteins are emerging at the confluence of maturations in technologies and large-scale biobanking efforts. In this perspective, we explore the current state of large-scale proteomic efforts in human blood, their common applications across disease and health phenotypes, as well as applications combining genomics. We also explore the challenges facing proteomics at biobank scale from wide-ranging considerations and provide a future outlook of the next steps the field may head towards.

The last 2 decades have seen rapid development in omics technology culminating in a combination of high-throughput methods to measure the genome, transcriptome and proteome at unprecedented scale (1). Genomics and transcriptomics are maturing to a stage where the vast majority of the entire genome or transcriptome can be ascertained through next generation DNA sequencing approaches. Dogmatically, the effects of genomic and transcriptomic changes principally culminate at the protein level, which constitutes the functional unit of biological processes. While proteomic technologies have made considerable advances, they lag behind genomics and transcriptomics in terms of overall coverage, cost and speed (2). That said, as described in this perspective, proteomics technologies in recent years have been carried out at a scale unthinkable just a few years ago.

**Fig. 1:**
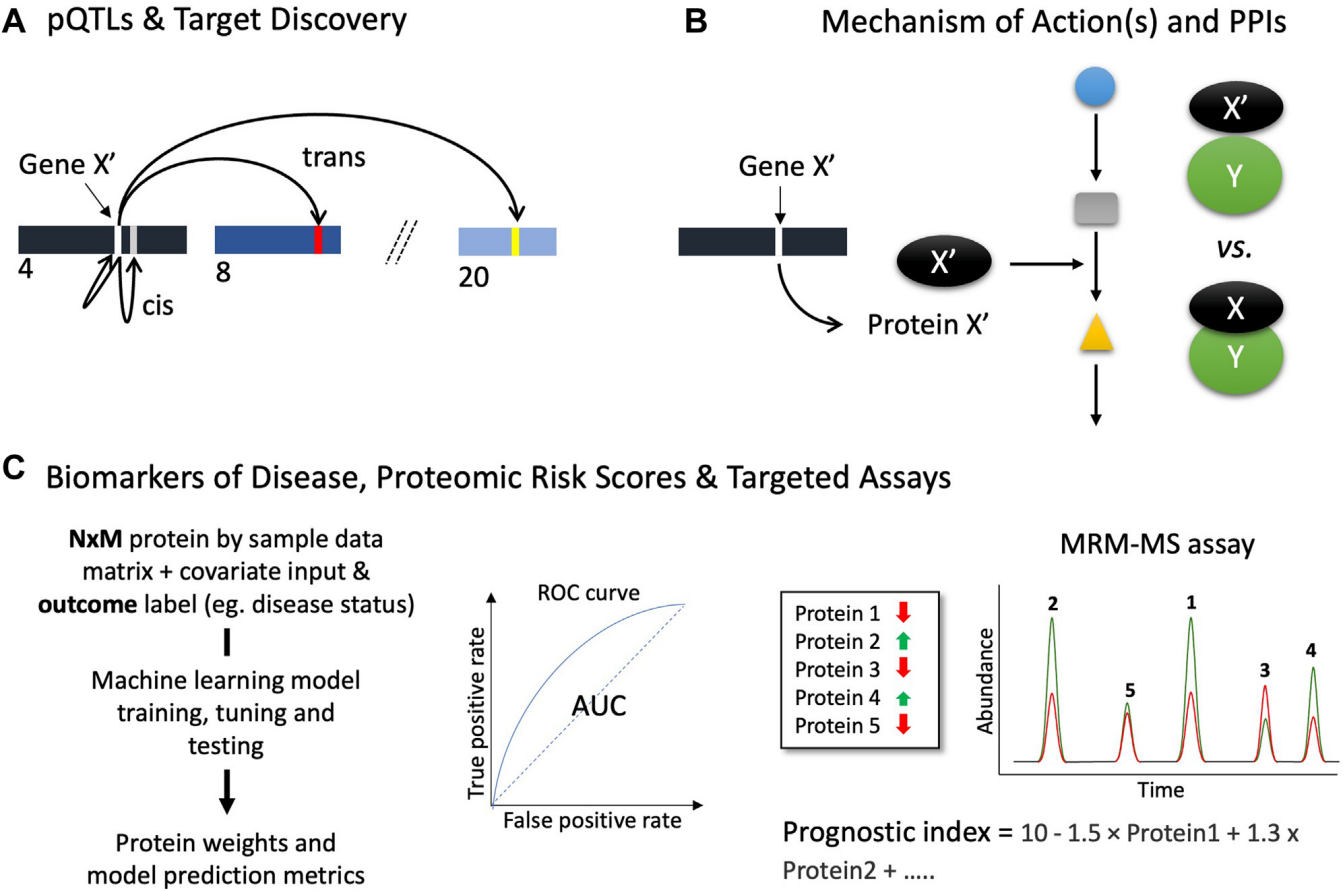
**Common uses of large-scale proteomics studies in blood.***A*, *cis* and *trans*-pQTLs and target discovery, (*B*) identifying possible mechanisms of action including altered activities and perturbations in protein-protein interactions, and (*C*) discovery of disease-specific biomarkers using machine learning, proteomic risk score analysis and MRM-MS peptide-based biomarker panel data to examine changes in relative abundances of one or more proteins between healthy and disease cohorts.

There are multiple reasons why industry and academic groups take on the immense costs and challenges of conducting large-scale population-based studies (see Fig. 1 and Box 1). Comprehensive data on the levels of blood proteins has many critical uses, perhaps none more important than identifying novel gene polymorphisms that can alter the concentrations of one or more blood proteins, otherwise referred to as *cis* and *trans* pQTLs (3). These gene-to-protein correlations can reveal novel biology, and in some cases become diagnostic biomarkers of disease or lead to the identification of novel therapeutic targets. In many cases, these data can help determine the mechanism of action of a gene and its encoded protein’s role in a particular disease process that, when mapped onto known biochemical pathways or protein-protein interaction networks, can also reveal novel up or downstream therapeutic targets and disease intervention strategies (4).

Box 1 **Main uses of population-based blood proteomics.**

**Table:** 

Main uses of population-based blood proteomics
- *Cis*- and *trans*-pQTL discovery - Novel therapeutic targets - Biomarkers of disease - Mechanisms of action - Disease risk prediction - Patient stratification for clinical trials

Unlike a specific tissue or cell type, proteins in the blood originate through multiple mechanisms, from ‘classical’ active secretion to shedding and cell leakage (5). It is not surprising that the major blood cells including erythrocytes, white blood cells, and platelets, along with the endothelial cells of the vascular system, are major contributors. In addition to these sources, the liver plays a major role in the active secretions of many blood proteins, although other organs and tissues throughout the body such as muscle also make significant contributions. Indeed, it has been postulated that there is a subset of unique proteins that can be tracked to every tissue or organ of the body and that this information can be used to generate disease-specific biomarkers (6).

While the human genome contains “only” 20,000 protein-encoding genes, the size of the proteome is much larger, with potential magnitude increase due to alternate forms through genetic variation and alternative splicing. In addition, there is a multitude of posttranslational modifications (PTMs), many of which have important regulatory functions, such as phosphorylation and glycosylation (7). Given that proteins may contain multiple PTMs in various combinations, there may be at least 10 million distinct proteoforms in the human proteome (8). Not all proteins are necessarily present in any one particular biological fluid or tissue at the same time, and the proteins that are present span a large dynamic range. This is especially true of blood proteins, which have a much larger dynamic range than the cellular proteome, with proteins ranging from high mg/ml abundances such as albumin and transferrin, to proteins involved in regulation and cell-cell signaling such as transcription factors, cytokines, and tumor-specific antigens that are often present at sub-pg/ml levels or observable only under certain pathological conditions (5). Complete capture of the entire proteome still falls short in coverage relative to genomics, but rapid progress in proteomic approaches has ushered in unprecedented insights from large-scale proteomics, especially in human blood plasma.

Although some large studies have used the serum, it is not surprising given the ease of collection, processing, and storage, blood plasma has been the most common fluid matrix employed in large proteomics studies in population cohorts/biobanks. There have been multiple reports examining preanalytical processing steps on analytical reproducibility including temperature, collection tube types, timing of centrifugations to length of storage. While each step can lead to variations in protein measurements, for plasma it is not surprising that the timing of the first centrifugation is most critical, as this key step removes blood cells that might otherwise degrade over time and release proteins that contaminate the plasma proteome (9). In any case, whether plasma, serum, or other sample types, it is critical that the methods employed for collection and processing across the entire cohort be rigorously adhered to, especially as the largest sample repositories are likely to span many years and even decades and often involve multiple collection sites (2, 10).

## Broad Overview of Proteomic Approaches

**Fig. 2:**
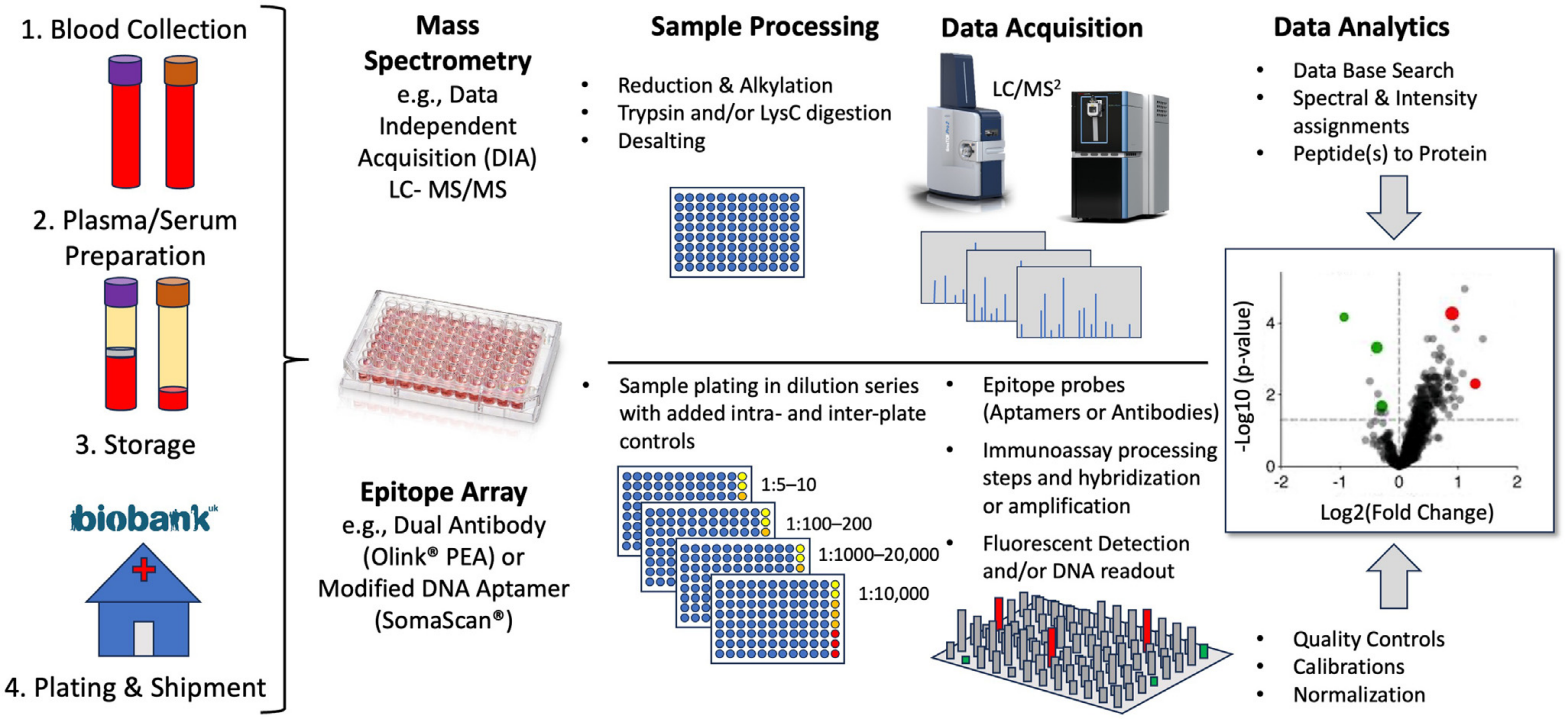
**Workflows for large-scale coverage of the serum or plasma proteome.** Mass spectrometry-based workflows that are currently capable of quantifying up to 2000 or more proteins is shown in the *top panel*. Highly multiplexed epitope-based array technology workflows such as Olink and SomaLogic are shown in the *bottom*. Both workflows require access to biobank (see far *left*, such as the UK Biobank) or clinical hospital banked serum or plasma specimens that require large-scale processing and storage facilities.

Current proteomics technologies can be categorized into two main approaches: mass spectrometry (MS) and affinity-based, both of which come in various forms. In affinity-based assays, protein-specific antibodies or aptamers are used to selectively bind to each target protein where the final readout is obtained using either fluorescence or DNA sequencing (11). This is typically carried out in sets of 96 or 384 well plate formats that can cover less than a hundred to several thousand unique proteins up to nine orders of magnitude in abundance. Most common platforms carry out various dilution steps to compensate for the large dynamic range in blood protein concentrations before a series of assay steps are carried out and final measurements are taken (see Fig. 2). Mass spectrometry methods on the other hand traditionally rely on the analysis of peptides generated by proteolytic digestion of the protein samples that are then separated by liquid chromatography and subjected to tandem mass spectrometry (LC-MS^2^) (see Fig. 2). There are many variations to the MS-based workflows, which can include depletion of high abundance proteins using multiplexed antigen removal columns (12), HPLC pre-fractionation of the peptide samples to generate separate pools of less complexity (13), multiplexed chemical labeling such as TMTpro (14), gas phase separation such as ion mobility (15), or nanoparticle enrichment (16). Most published MS protocols share most steps and ultimately rely on various commercial and academic software suites to make the final spectral assignments. For the newer data-independent acquisition (DIA) workflows that many believe to be best suited for large-scale plasma proteomics studies due to their superior acquisition speeds required for large-scale studies, the DIA software platforms such as Spectronaut, DIA-NN, MaxDIA, and Skyline have significantly improved data processing speed with increased proteome coverage and are the most widely used in these types of studies (17).

## Emergence of soluble proteomics in populational cohorts

**Fig. 3:**
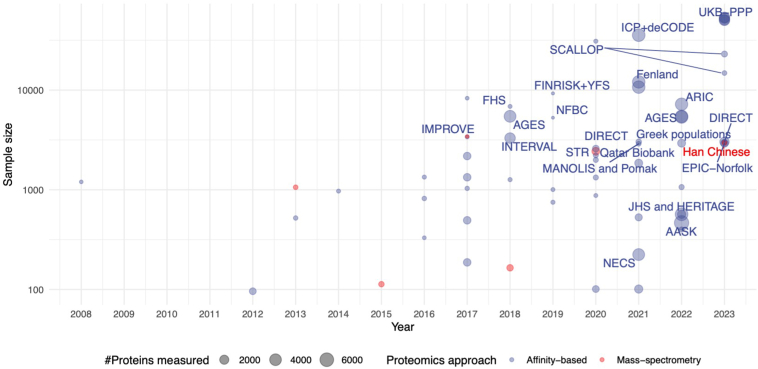
**Sample size and proteomic coverage of human plasma proteogenomic studies between 2008 and 2023.** Studies with sample sizes >2500 or >2000 proteins measured are labelled. *Red* indicates MS-based proteomics, *blue* indicates affinity-based proteomics. MANOLIS and Pomak are part of the HELIC (HELlenic Isolated Cohorts study) cohort.

Maturation and advances in these populational cohorts/biobanks and proteomic technologies have ushered in significant increases in both sample sizes and proteins assayed, as exemplified by how proteogenomic studies have evolved over the last decade or so (Fig. 3). Cohorts with sample sizes beyond 10,000 measuring more than 1000 proteins are beginning to emerge more frequently since 2020 (4, 18, 19), with the vast majority of studies still predominantly focusing on Caucasian populations. In large scale (>5000) population sized cohorts, affinity-based methods, largely due to their high-throughput characteristics and cost considerations, are currently the predominant approach. Recent proteomics studies in biobank-scale cohorts such as deCODE (19) and UKB-PPP (4) have demonstrated feasibility of affinity-based proteomics at tens of thousands of samples measured over a span of months, paving the way for potential future scale-up to entire biobank sizes of >100,000 samples, currently prohibitive for MS methods.

As mentioned earlier, despite recent improvements in both throughput and sensitivity, MS-based proteomics still lags behind epitope-array-based technologies in the throughput needed for routine measurements beyond thousands of samples as well as the inherently more limited dynamic range needed to detect and quantify proteins at lower concentrations (<ng/ml). An effort at the scale of UKB-PPP (∼50,000 samples) would likely have taken several years or a very high degree of platform multiplexing with standard MS set-ups available to us at the start of that effort in 2021 and would likely not have captured many low abundance blood proteins, some of which are well known to be directly linked to specific disease states. In part, this is attributable to the inherently more complex workflow and processing pipeline of intermediate data generated by MS methods, which can introduce technical variation as well as being time consuming, as opposed to the relatively simpler multiplexing and automatable set-up of affinity-based methods. Despite this, MS studies in thousands of individuals (including non-Europeans) with and without genomic information are beginning to emerge (20, 21).

Unlike genomics data, the size of current affinity-based proteomics data generated in populational cohorts is relatively modest in comparison. Taking UKB-PPP as an example, in its simplest form, the data is essentially a matrix of ∼50,000 individuals by ∼3000 protein analytes, taking up approximately 3 GB of storage which can feasibly run locally. In comparison, the same type of bulk transcriptomic data (∼18,000 gene transcripts) is ∼6 times larger, and genomic data (without compression) measuring 12 million variants is ∼4000 times larger, requiring higher order storage and processing capabilities in the form of cluster or distributed cloud computing. Even with the recent expansion of the Olink and SomaLogic assay coverage to between 5400 and 10,000 unique human proteins respectively, the size remains relatively modest. On the other hand, MS proteomics can generate potentially comparably large data to genomics given the more complex intermediate and peptide level data, which will also require significant aggregation and processing to get to much smaller protein level abundance read-outs per sample. For instance, the dataset generated using the Seer Proteograph platform in a recent study with 345 samples extended to 4 TB of spectral data, which is comparable in size to the sequencing data produced by whole genome sequencing of a similar-sized cohort (16).

## Combining Proteomic and Genomic Data for the Understanding of Disease

Availability of large populational scaled biobanks and cohorts with genomics and proteomic information has ushered in studies mapping genetic associations with soluble protein abundances (protein quantitative trait loci, pQTLs) akin to the analogous studies in gene expression (eQTLs) (3) – a comprehensive up-to-date list of pQTL studies is maintained at http://www.metabolomix.com/a-table-of-all-published-gwas-with-proteomics/. These studies have progressively increased in sample size and proteins measured and are extending beyond blood plasma to other tissue matrices such as cerebrospinal fluid (Fig. 3, Table 1 provides a current snapshot focused on blood proteomics pQTL studies). The increasing availability of pQTL studies has greatly enhanced our understanding of genetic architecture regulating plasma protein abundances in terms of heritability, local (*cis*) and distant (*trans*) are enabling genetic-based inference of proteome in large cohorts genetic architectures with insights into pathways regulating protein levels and protein-protein interactions, causal insights into potential protein targets/pathways driving disease risk through approaches such as colocalization and Mendelian randomization with implications for drug targeting and safety indications. Recently, genetic scores have been constructed from large sample sizes for molecular omics traits including proteomics without such information to generate putative insights before embarking on often costly and time-consuming data generation (22). It is also important to note that genetic-based approaches provide static imputation of average protein (and other omic) levels covering only the heritable component. The proteome is dynamic and changes depending on alterations in context (*e.g.*, environment, lifestyle, physiology, infection)—which are not captured in static genomic information. Ways of capturing the non-genetic variation of the proteome using epigenomics have been recently suggested (23, 24, 25).

**Table 1:** Snapshot of blood-based proteomics pQTL studies

Reference	Ancestry	Platform	# Samples	# Proteins	# pQTLs	Study notes	URL
Melzer *et al.* (PLoS Genet, 2008)	European	Immuno	1200	42	8	Population study	https://www.ncbi.nlm.nih.gov/pubmed/18464913
Lourdusamy *et al.* (Hum Mol Genet, 2012)	European	SOMA	96	778	60	Elderly Europeans	https://www.ncbi.nlm.nih.gov/pubmed/22595970
Johansson *et al.* (PNAS, 2013)	European	MS	1060	163	5	Two population cohorts	https://www.ncbi.nlm.nih.gov/pubmed/23487758
Kim *et al.* (PLoS One, 2013)	European	Immuno	521	132	28	Altzheimer's disease cohort	https://www.ncbi.nlm.nih.gov/pubmed/23894628
Enroth *et al.* (Nature Comm, 2014)	European	Immuno	970	77	18	Population study	https://www.ncbi.nlm.nih.gov/pubmed/25147954
Liu *et al.* (Mol Syst Biol, 2015)	European	MS	113	342	18	Female twins	https://www.ncbi.nlm.nih.gov/pubmed/25652787
Sun *et al.* (PLOS Genet, 2016)	European	Immuno	1340	88	527	Current and former smokers	https://journals.plos.org/plosgenetics/article?id=10.1371/journal.pgen.1006011
Deming *et al.* (Sci Rep, 2016)	European	Immuno	818	146	56	Alzheimer cohort	https://www.ncbi.nlm.nih.gov/pmc/articles/PMC4698720/
Solomon *et al.* (Circ Cardiovasc Genet, 2016)	European	Immuno	330	51	27	Population study	https://www.ncbi.nlm.nih.gov/pubmed/27329291
Ahola-Olli *et al.* (Am J Hum Genet, 2017)	European	Immuno	8293	48	27	Population study	https://www.ncbi.nlm.nih.gov/pubmed/27989323
Di Narzo *et al.* (PLoS Genet, 2017)	European	SOMA	187	1128	41	Inflammatory bowel disease patients	https://www.ncbi.nlm.nih.gov/pubmed/28129359
Suhre *et al.* (Nature Comm, 2017)	European	SOMA	1335	1124	539	KORA and QMDiab study (Germany and Qatar)	https://www.nature.com/articles/ncomms14357
Folkersen *et al.* (PLoS Genet., 2017)	European	Immuno	3394	83	79	Population study	https://www.ncbi.nlm.nih.gov/pubmed/28369058
de Vries *et al.* (Hum. Mol. Genet., 2017)	European + African American	MS	3424	25	22	European and African Americans (ARIC study)	https://academic.oup.com/hmg/article/26/17/3442/3953984
Ahsan *et al.* (PLoS Genet, 2017)	European	OLINK	1033	121	45	Population study	http://journals.plos.org/plosgenetics/article?id=10.1371/journal.pgen.1007005
Carayol *et al.* (Nature Comm, 2017)	European	SOMA	494	1129	55	Obese subjects	https://www.nature.com/articles/s41467-017-02182-z
Benson *et al.* (Circulation, 2017)	European	SOMA	2180	1129	161	Framingham Heart Study and Malmo Diet and Cancer Study	https://doi.org/10.1161/CIRCULATIONAHA.117.029536
Sun *et al.* (Nature, 2018)	European	SOMA	3301	2994	1927	Interval study (UK blood donors)	https://www.nature.com/articles/s41586-018-0175-2
Emilsson *et al.* (Science, 2018)	European	SOMA	5457	4137	3134	AGES Reykjavik study (Islanders over 65)	http://science.sciencemag.org/content/early/2018/08/01/science.aaq1327
Yao *et al.* (Nature Comm, 2018)	European	Immuno + SOMA	6861	71	105	Framingham Heart Study	https://www.nature.com/articles/s41467-018-05512-x
Zhernakova *et al.* (Nature Genet 2018)	European	OLINK	1264	92	214	LifeLines Dutch population cohort	https://www.nature.com/articles/s41588-018-0224-7
Solomon *et al.* (Circulation, 2018)	European	MS	165	664	60	Tromsö Study	https://www.ahajournals.org/doi/10.1161/CIRCGEN.118.002170
Sliz *et al.* (J Med Genet, 2019)	European	Immuno	5284	16	16	Northern Finland Birth Cohort 1966 + meta-analysis	https://jmg.bmj.com/content/early/2019/06/19/jmedgenet-2018-105965
Hillary *et al.* (Nat Comm, 2019)	European	OLINK	750	92	41	Lothian Birth Cohort 1936	https://www.nature.com/articles/s41467-019-11177-x
Höglund *et al.* (Sci Rep, 2019)	European	OLINK	1005	72	18	Northern Swedish population health study (NSPHS)	https://www.nature.com/articles/s41598-019-53111-7
Nath *et al.* (AJHG 2019)	European	Immuno	9267	18	8	Three population-based cohorts	https://www.cell.com/ajhg/fulltext/S0002-9297(19)30389-1
Folkersen *et al.* (Nature Metabol, 2020)	European	OLINK	30,931	90	451	SCALLOP consortium, incl. 13 studies	https://www.nature.com/articles/s42255-020-00287-2
Sjaarda *et al.* (AJHG, 2020)	Latin American	Immuno	2216	237	46	Latin Americans, ORIGIN study	https://www.cell.com/ajhg/fulltext/S0002-9297(20)30016-1
Gilly *et al.* (Nat Comm, 2020)	European	OLINK	1328	257	131	Hellenic Isolated Cohorts MANOLIS study	https://www.nature.com/articles/s41467-020-20079-2
Zhong *et al.* (BMC Genome Med, 2020)	European	OLINK	101	794	144	Longitudinal wellness cohort from Sweden	https://genomemedicine.biomedcentral.com/articles/10.1186/s13073-020-00755-0
Dodig-Crnkovic *et al.* (EBioMedicine, 2020)	European	Immuno	2592	734	15	Swedish Twin Registry, born 1911–1958	https://www.sciencedirect.com/science/article/pii/S2352396420302292
Hillary *et al.* (Genome Medicine, 2020)	European	OLINK	876	70	13	Lothian Birth Cohort 1936	https://genomemedicine.biomedcentral.com/articles/10.1186/s13073-020-00754-1
Ruffieu *et al.* (PLoS Comp Bio 2020)	European	MS + SOMA	2433	1230	136	Optifast Canadian & DiOGenes cohort	https://journals.plos.org/ploscompbiol/article?id=10.1371/journal.pcbi.1007882
Bretherick *et al.* (PLoS Genet, 2020)	European	OLINK	1992	249	154	Isolated populations from Orkney (Scotland) and Vis (Croatia)	https://journals.plos.org/plosgenetics/article?id=10.1371/journal.pgen.1008785
Pietzner *et al.* (Nature Comm, 2021)	European	SOMA	10,708	4775	220	Fenland study (UK)	https://www.nature.com/articles/s41467-020-19996-z
Zhong *et al.*, (Nature Comm, 2021)	European	OLINK	101	1463	331	Longitudinal wellness cohort from Sweden	https://www.nature.com/articles/s41467-021-22767-z
Gurinovich *et al.* (GeroScience, 2021)	European	SOMA	224	4131	21	New England Centenarian Study (NECS)	https://link.springer.com/article/10.1007/s11357-021-00376-4
Yang *et al.* (Nature Neuro, 2021)	European	SOMA	529	931	127	Europeans with and without Alzheimer's disease	https://www.nature.com/articles/s41593-021-00886-6
Pietzner *et al.* (Science, 2021)	European	SOMA	12,084	4775	10674	Fenland study	https://www.science.org/doi/10.1126/science.abj1541
Katz *et al.* (Circulation, 2021)	African American	SOMA	1852	1301	569	Black adults (Jackson Heart Study)	https://www.ahajournals.org/doi/10.1161/CIRCULATIONAHA.121.055117
Ferkingstad *et al.* (Nature Genet, 2021)	European	SOMA	35,559	4907	18084	Icelanders	https://www.nature.com/articles/s41588-021-00978-w
Png *et al.* (Nat Comm, 2021)	European	OLINK	2893	184	214	MANOLIS and Pomak, part of the Hellenic Isolated Cohorts (HELIC)	https://www.nature.com/articles/s41467-021-27387-1
Macdonald-Dunlop *et al.* (medRxiv, 2021)	European	OLINK	26,494	184	1308	18 cohorts of European ancestry from Scallop	https://www.medrxiv.org/content/10.1101/2021.08.03.21261494v1
Emilsson *et al.* (Nat Comm, 2022)	European	SOMA	5343	4782	5451	AGES study (Islanders over 65)	https://www.nature.com/articles/s41467-022-28081-6
Gudjonsson *et al.* (Nat Comm, 2022)	European	SOMA	5368	4782	4113	AGES study (Islanders over 65)	https://www.nature.com/articles/s41467-021-27850-z
Katz *et al.* (Science Advances, 2022)	European	SOMA + OLINK	787	6451	798	Jackson Heart Study and HERITAGE Family Study	https://www.science.org/doi/10.1126/sciadv.abm5164
Zhang *et al.* (Nature Genet, 2022)	European	SOMA	7213	4657	1384	Atherosclerosis Risk in Communities (ARIC) cohort study	https://www.nature.com/articles/s41588-022-01051-w
Zhang *et al.* (Nature Genet, 2022)	African American	SOMA	1871	4657	1005	Atherosclerosis Risk in Communities (ARIC) cohort study	https://www.nature.com/articles/s41588-022-01051-w
Thareja *et al.* (Human Mol Genet, 2022)	Arabs	SOMA	2935	1301	2685	Qatar Biobank	https://academic.oup.com/hmg/advance-article/doi/10.1093/hmg/ddac243/6724969
Surapaneni *et al.* (Kidney International, 2022)	African American	SOMA	466	6790	696	African Americans	https://www.kidney-international.org/article/S0085-2538(22)00547-6/fulltext
Caron *et al.* (Genome Medicine, 2022)	European	Immuno	400	229	152	Milieu Interieur cohort, France	https://genomemedicine.biomedcentral.com/articles/10.1186/s13073-022-01032-y
Hillary *et al.* (Alzheimers Dement, 2022)	European	SOMA	1064	282	64	Scottish Family Health Study	https://alz-journals.onlinelibrary.wiley.com/doi/10.1002/dad2.12280
Koprulu *et al.* (Nature Metabolism, 2023)	European	OLINK	2923	1180	256	EPIC-Norfolk (UK)	https://www.nature.com/articles/s42255-023-00753-7
Brown *et al.* (Nat Comms, 2023)	European	OLINK	3029	373	2123	DIRECT study	https://www.nature.com/articles/s41467-023-40569-3
Niu *et al.* (medRxiv, 2023)	European	MS	1914	420	712	HOLBAEK Study, children and adolescents (Denmark)	https://www.medrxiv.org/content/10.1101/2023.03.31.23287853v1
Xu *et al.* (Nat Comm, 2023)	Chinese	MS	2958	304	195	Han Chinese	https://www.nature.com/articles/s41467-023-36491-3
Zhao *et al.* (Nat Immun, 2023)	European	OLINK	14,824	91	180	11 cohorts from the Scallop consortium	https://www.nature.com/articles/s41590-023-01588-w
Sun *et al.* (Nature 2023)	European	OLINK	54,219	2923	14287	UK Biobank Pharma Proteomics Project	https://www.nature.com/articles/s41586-023-06592-6
Dhindsa *et al.* (Nature 2023)	European	OLINK	49,736	2923	5433	UK Biobank Pharma Proteomics Project	https://www.nature.com/articles/s41586-023-06547-x
Eldjarn *et al.* (Nature 2023)	European	OLINK	46,218	2941	26926	UK Biobank Pharma Proteomics Project	https://www.nature.com/articles/s41586-023-06563-x
Carland *et al.* (Clin Proteom, 2023)	European	OLINK	22,997	92	503	12 cohorts of primary European ancestry from Scallop	https://clinicalproteomicsjournal.biomedcentral.com/articles/10.1186/s12014-023-09421-0
Gilly *et al.* (Molecular Metabolism, 2023)	European	OLINK	3005	92	322	Two isolated Greek populations	https://doi.org/10.1016/j.molmet.2023.101810
Said *et al.* (medRxiv, 2023)	Chinese	OLINK	3974	1451	2872	China Kadoorie Biobank	https://www.medrxiv.org/content/10.1101/2023.11.13.23298365v1
Suhre *et al.* (Cell Genomics 2024)	European	OLINK	52,705	2821 ratios	8462	UK Biobank Pharma Proteomics Project	https://www.cell.com/cell-genomics/fulltext/S2666-979X(24)00033-8

## Applications of Proteomics for Disease Prediction

The emergence of large multiplex proteomic data in populational cohorts is beginning to usher in unprecedented exploration in proteomics-based predictive models beyond traditional risk factors based on a limited set of biomarkers or proteins. Using traditional penalized regression models, multiple studies have shown the utility of multiplex proteomics in predicting demographic factors such as age (4, 26, 27), body mass index (4, 28), renal function (4, 29), health metrics (29) as well as disease (30, 31, 32).

Proteomic prediction models also facilitate the development of risk scores akin to polygenic risk scores for a multitude of diseases (33). Whilst the score training and testing are fundamentally not dissimilar, there are some unique differences in developing proteomic *versus* genetic risk scores – proteomic data is magnitude narrower than genomic data and thus has less model under specification limitations where the number of predictors greatly exceeds sample size; certain proteome levels undergo dynamic changes as the disease progresses or at disease onset and may also be influenced by a range of non-causal factors as opposed to the germline genetic variation that are invariably unchanged throughout life. It can be envisaged that a combined omics score may offer additional risk prediction benefits than any score alone, especially in light of the relatively low-risk variance explained for many disease genetic risk scores. Lastly, in view of potential drops in genetic risk score performance across ancestries (34), the portability of proteomic risk models across populations and ancestry remains to be estimated and are important validation steps for generalizable models.

## Challenges and Limitations

Despite significant advances in the field, there remains a range of challenges that need to be addressed both from technical and scientific viewpoints as we look towards the horizon for future outlook and opportunities—summarized in Box 2 and expanded below.

Box 2 **Summary of challenges and future outlook of populational proteomics.**

**Table:** 

Challenges and limitations	Future outlook and opportunities
- Limited correlation between assay technologies - Distinguishing between technical and biological variation - Orthogonal validation using multiple platforms - Reliable quantification that is transferable between studies - Multiple testing considerations, especially with smaller scale clinical studies	- Expected technological advances - Post-translational modifications - Other tissue types at scale - Proteomics in diverse ancestries - Longitudinal and physiological proteomic changes - Combining evidence across studies - Data sharing and legal considerations

### Limited Correlation Between Assay Technologies

Due to practical and organizational considerations, many populational cohorts have predominantly adopted one large-scale proteomics approach, largely affinity-based. However, the same protein measurement may differ between proteomic assay approaches and this would have an impact in the transferability and generalizability of proteomic models across technology. A recent head-to-head study between SomaScan and Olink showed a range of poor to high Spearman’s correlation for overlapping proteins with a median from studies ranging from 0.20-0.44 (35, 36, 37) whilst in a study comparing three affinity-based platforms in a COVID-19 cohort showed median Kendell correlation ranging 0.36 to 0.70 between those platforms (38). For proteins with disparaging measures, it is difficult to establish one as more accurate than the other in the absence of gold standards. A wide range of factors may contribute to the lack of correlation such as reagents binding to different forms of the target, interactions with other proteins/complexes/materials in blood, sensitivities to protein abundances etc. This problem would be expected to be more pronounced for assays targeting the lowest abundance blood proteins, contributing to larger CVs - as observed in the recent UKB-PPP study (4) - and potentially a higher likelihood of erroneous measurements due to off target cross reactivities. From the selection of candidates measured with the traditional ELISA approach, the Olink approach seem to concord better with ELISAs than SomaScan (35, 36), which may reflect Olink’s use of antibodies, like ELISAs, as the capture reagents as opposed to aptamers. Noteworthy here is also a recent comparison of the Olink and the Nulisa platforms with a multiplexed cytokine assay, demonstrating good pairwise correlations of the three platforms (39). In addition to differences within affinity-based proteomic assays, there may also be systematic differences between affinity-based and MS approaches that may be due to significant differences in technology and sample processing steps prior to measurement with the instruments (40).

### Distinguishing Between Technical and Biological Variation

There are also important distinctions between technical and biological reproducibility as opposed to concordance across platforms across studies. Both assays have been shown in multiple studies to have good reproducibility in terms of relatively low coefficients of variation and biological associations of the same assay approach across studies (4, 18, 35, 36). It is important to note that models and associations derived from one approach may generalize less well across assay platforms compared to within.

### Orthogonal Validation

Most large-scale studies, including both mass spectrometry and epitope scanning technologies, often include a few examples where an orthogonal approach is used to provide independent validation of a handful of proteins. This is largely a ‘trust but verify’ approach to large-scale discovery experiments such as using ELISAs to validate specific high-plex proteomic findings (35, 36). However, ELISAs are not well suited for many targets due to interferences and typically lack multiplexing capabilities for a set of custom targets. Multiple reaction monitoring (MRM) mass spectrometry is the preferred ’gold standard’ in these cases where stable isotope-encoded peptides are also employed as internal standards (41). This type of independent validation is deemed essential if one is to construct a clinical biomarker assay. That said, MRM-MS assays can take time and resources to set up properly, especially if stable isotope peptides are employed and the response curves are properly determined over a broad dynamic range. Fortunately, constructing and validating large sets of quantitative peptide-based assays using MRM-MS is becoming faster and less expensive due to the availability of synthetic peptide reagents (42) and more sensitive triple quadrupole instruments (41).

### Absolute *versus* Relative Quantification

Owing to various technical and practical constraints, large-scale wide proteomic measures are quantified on relative rather than absolute scales. For investigations such as pQTL mapping and associations looking for statistically significant effects without necessitating absolute effect sizes, relative quantification has been sufficient. However, absolute quantification offers additional value in having a more interpretable effect size and allows for calibration and comparability across studies which is extremely difficult across studies without bridging samples or making assumptions about the underlying samples. Operating on relative quantifications may create additional challenges in calibrating measures in additionally measured samples cross-sectionally and longitudinally as well as harmonizing across datasets/populations. Absolute quantification is also important in standardized reference ranges in the clinical setting as values need to be comparable across runs and centres.

### Multiple Testing Considerations

Following in the direction of genomics, there needs to be additional scrutiny in accounting for multiple testing, akin to the candidate gene *versus* genome-wide testing era, to minimize false positives in proteomic association findings. For example, assessing one protein from a proteomic array and finding an association at nominal *p* < 0.05 for one outcome in a biobank cohort without replication data, is by far too lenient and may lead to replication difficulties for many findings. However, the multiple correction threshold can be challenging to set, since one may argue to consider the number of independent proteins tested, or the entire human proteome of ∼20,000, or whether to consider potential isoforms and post-translational modifications which brings up the potential testing space significantly. Additional empirical studies similar to the genomics field will be needed to determine the optimal analogous “proteome-wide significance” with emphasis on independent replication recommended. Sharing of full summary statistics in an easily accessible manner is increasingly more important to maintain consistency and further downstream utilization of these results.

## Future Outlook and Opportunities

### Expected Technological Advances

Given the continued improvement in coverage and throughput, coupled with a reduction in cost, it is inevitable that studies with larger sample sizes and increased proteome coverage will emerge to further empower proteomic association discovery both from proteogenetic and non-genetic perspectives. Indeed, some of the newer proteomic technologies based on spatial imaging, nanopores or Edman-like sequential degradation could well represent a breakthrough should they succeed in both speed and high multiplexing needed to for blood-based applications to surpass or be competitive with current technologies (43). Some of these newer technologies are also capable of single molecule detection, *i.e.*, proteins or peptides, that promise unparalleled sensitivity as well as absolute quantification (44, 45). SomaLogic and Olink epitope array platforms are likely to continue to increase their protein coverage as they have over the last few years, although it is not clear at what point they will reach a plateau due to specificity and accuracy limitations as they target lower abundance blood proteins. In the MS realm, recent innovations in next-generation mass spectrometry platforms that have incorporated time-of-flight (TOF) analyzers in combination with orbitrap analyzers or trapped ion mobility spectrometry (TIMS) have significantly reduced the time needed for plasma analysis without sacrificing depth or coverage (46, 47). A recent example of such an approach employed a timsTOF MS analyzer along with a prior separation step utilizing a novel nanoparticle peptide enrichment scheme to successfully map over a hundred novel pQTLs (16). Indeed, the Orbitrap Astral and timsTOF Ultra from Thermo Scientific and Bruker respectively, are now becoming competitive with array-based methods in terms of increased sensitivity, comprehensive detections, and speed (16) (https://www.genomeweb.com/proteomics-protein-research/mass-spec-retakes-center-stage-2023-next-gen-proteomics-startups).

### Post-translational Modifications

The existence of multiple proteoforms for any given protein presents serious and largely unmet challenges. Protein ratios and other combinations extend the permutation space even further (48). Splice variants, single amino acid polymorphisms, and PTMs such as phosphorylation and glycosylation, together can greatly expand the ensemble of proteins in the blood, as well as lead to significant differences among individuals or disease states. For antibody and aptamer screening platforms, this can make accurate detection difficult due to the occlusion or alterations in binding efficiencies to their target epitopes. Mass spectrometry methods have the potential to more readily identify and differentiate among proteoforms but require high peptide coverage or additional PTM-based enrichment steps to be highly effective. Progress in both these methodologies may eventually circumvent these current limitations, or alternatively, some of the newer proteomic technologies that rely on single protein or peptide detection may be better suited for this challenge. New insights may also come from more specialized technologies that target PTM directly, such as hydrophilic interaction ultra-performance liquid chromatography (HILIC-UPLC) to fine-map the human blood plasma N-glycome (49).

### Other Tissue Types at Scale

While large-scale proteomics studies have been almost exclusively in plasma or serum, there are clearly other bodily fluid sources such as CSF, saliva, and urine (50, 51), or non-liquid sources such as tumors, organ biopsies, or formalin-fixed paraffin-embedded (FFPE) clinical samples (52), that offer alternative and perhaps superior information for specific diseases. Additionally, pQTL mapping studies have been performed at these smaller scales in non-blood-based contexts (53, 54, 55, 56, 57, 58, 59, 60, 61, 62) with potential larger studies emerging (63). In most non-liquid sample types, MS-based proteomics has the current advantage in coverage and adaptability and will likely be the main technology driving these types of studies. Exploiting the large historical collection of FFPE samples seems especially attractive given their clinical relevance, and one would hope to see more of these types of sample sets being analyzed as newer high-throughput MS workflows become more widely utilized (64, 65).

### Proteomics in Diverse Ancestries

Akin to the genomics field, the majority of large-scale proteomics studies have focused on European ancestries. Given the genetic and environmental diversities that may affect protein abundances, studies in other ancestry groups and regions will be highly informative in elucidating these effects with some biobank efforts emerging (37). Studies in other ancestry groups will also shed light on environmental and behavioral impacts on protein levels as well as improve the generalizability of proteomics risk scores.

### Longitudinal and Physiological Proteomic Changes

In addition to disease states affecting protein abundances either acutely and/or transiently or chronically, certain proteins may be highly dynamic longitudinally in normal physiological states. Large-scale studies to date have mostly focused on cross-sectional snapshots of protein levels with variable time before or after disease as a result of sampling rather than by design. Longitudinal measures so far have been understudied; however, dynamic protein changes can be challenging to capture as variations occur on the scale ranging from hours in circadian patterns to months throughout the year influenced by environmental and seasonal changes and even years at different stages in life. There is also additional complexity arising of physiological differences between sex at various stages in life such as puberty, pregnancy, and menopause. Therefore, the frequency at which longitudinal samples need to be ascertained remains a challenge and is currently limited by the availability of longitudinal samples.

### Combining Evidence Across Studies

Sharing or merging multiple proteomic data sets would clearly benefit the scientific community and lead to more rapid progress in identifying novel pQTLs and disease biomarkers. There are many difficulties to overcome including normalization and comparability across independent studies, especially if different proteomic platforms are being used. One partial solution to this problem was the formation of the SCALLOP consortium (http://www.scallop-consortium.com/) where academic users, now encompassing over 28 research institutions, share plasma proteomic data analyzed using Olink’s platform primarily for pQTL discovery (66). More recently, an industry-centered SCALLOP initiative has been proposed for analyzing longitudinal plasma or serum samples in the control or placebo arms of clinical trials using the Olink and potentially other proteomic platforms (personal communication, Anders Mälarstig). Should these and other such collaborations become more common, one would expect to see exponential growth in the volume of publicly available blood-based proteomic data.

### Data Sharing and Legal Considerations

Lastly, some of the most challenging aspects of designing and implementing large-scale proteomics studies are not necessarily technical, but rather have financial, organizational, contractual, data access, and intellectual property implications. In the UKB-PPP consortium, the alignment of multiple pharmaceutical companies necessitated legal departments with sometimes conflicting objectives to negotiate among the consortium members, and then with both the UKB as the gatekeeper of the large bank of plasma samples and Olink as the technology and data provider to reach agreements on sample selection and handling, timing, and the crucial period of restricted data access before it was to be made public. As we expect to see even larger or more complex studies undertaken in the coming years, the logistical challenges of sample procurement alone will likely require the participation of several biobanks and hospital resources to increase the study's power and ethnic diversity. Funding such studies also pose serious challenges, and one could imagine that joint government and industry participation, along with nonprofit organizations, would be needed to bring the proper resources for such large-scale multiple-year initiatives. Finding proper solutions to these many technical and other challenges will hopefully lead to a more widely accessible proteomic database that will benefit private, industry, and government-funded scientists alike.

## Data Availability

All data are contained within the manuscript.

## Conflict of interest

The authors declare the following financial interests/personal relationships which may be considered as potential competing interests:

BBS is an employee of Bristol Myers Squibb and former employee of Biogen.
